# A genome-wide association study identifies multiple loci associated with mathematics ability and disability

**DOI:** 10.1111/j.1601-183X.2009.00553.x

**Published:** 2010-03

**Authors:** S J Docherty, O S P Davis, Y Kovas, E L Meaburn, P S Dale, S A Petrill, L C Schalkwyk, R Plomin

**Affiliations:** † Social, Genetic and Developmental Psychiatry Centre, Institute of Psychiatry, King's College LondonUK; ‡ University of New Mexico, Department of Speech & Hearing SciencesUSA; § Center for Developmental and Health Genetics, The Pennsylvania State UniversityUSA

**Keywords:** DNA pooling, quantitative trait, allelic association, SNP microarrays, mathematical ability, cognitive traits

## Abstract

Numeracy is as important as literacy and exhibits a similar frequency of disability. Although its etiology is relatively poorly understood, quantitative genetic research has demonstrated mathematical ability to be moderately heritable. In this first genome-wide association study (GWAS) of mathematical ability and disability, 10 out of 43 single nucleotide polymorphism (SNP) associations nominated from two high- vs. low-ability (*n* = 600 10-year-olds each) scans of pooled DNA were validated (*P* < 0.05) in an individually genotyped sample of *2356 individuals spanning the entire distribution of mathematical ability, as assessed by teacher reports and online tests. Although the effects are of the modest sizes now expected for complex traits and require further replication, interesting candidate genes are implicated such as *NRCAM* which encodes a neuronal cell adhesion molecule. When combined into a set, the 10 SNPs account for 2.9% (*F* = 56.85; df = 1 and 1881; *P* = 7.277e–14) of the phenotypic variance. The association is linear across the distribution consistent with a quantitative trait locus (QTL) hypothesis; the third of children in our sample who harbour 10 or more of the 20 risk alleles identified are nearly twice as likely (OR = 1.96; df = 1; *P* = 3.696e–07) to be in the lowest performing 15% of the distribution. Our results correspond with those of quantitative genetic research in indicating that mathematical ability and disability are influenced by many genes generating small effects across the entire spectrum of ability, implying that more highly powered studies will be needed to detect and replicate these QTL associations.

Mathematics is fundamental to many fields such as science, engineering, economics and medicine, and the understanding of basic numeracy and related skills is a crucial component of normal brain function. Despite widespread appreciation of the increasing importance of numeracy in modern society, research has revealed poor average performance in many countries, with extremely low enrolment in mathematical subjects after age 16 (Mazzocco & Myers 2003; Smith 2004). Defining disability in mathematics rests on establishing a cut-off, which can be performed in a variety of ways. One approach defines disability as obtaining arithmetic scores at least 2 years below expected grade level (American Psychiatric Association 1994). With this definition, mathematical disability has an estimated frequency of 6% in school children (Gross-Tsur *et al.* 1996), a prevalence similar to that of reading disability (Law *et al.* 1998). Understanding the etiology of mathematical ability and disability may prove an essential step in tackling mathematical underachievement, and could provide fresh insights into human brain function.

Quantitative genetic research indicates a genetic component to individual variation in mathematical ability, yielding heritability estimates of 0.2–0.9 (Alarcón *et al.* 2000; Husén 1959; Kovas *et al.* 2007a,b; Light *et al.* 1998; Loehlin & Nichols 1976; Oliver *et al.* 2004; Thompson *et al.* 1991; Wadsworth *et al.* 1995). In the absence of obvious neurological impediment mathematical disability is a complex disorder. As with variation in normal levels of mathematical ability, quantitative genetic studies have attributed a similar level of genetic influence to mathematical disability (Alarcón *et al.* 1997; Kovas *et al.* 2007a,b; Oliver *et al.* 2004). Importantly, quantitative genetic findings also suggest that rather than being a distinct clinical category, mathematical disability is the quantitative extreme of the normal distribution of ability—influenced by many of the same genetic factors affecting normal variation in ability (Alarcón *et al.*, 1997; Kovas *et al.* 2007a,b; Oliver *et al.* 2004). This supports a quantitative trait locus (QTL) approach to the molecular genetic study of mathematical ability and disability (Plomin *et al.*, 2009).

At present no molecular genetic research specifically investigating mathematical ability or disability has been reported. With linkage approaches lacking the power required to detect the small effects expected in complex traits (Plomin *et al.* 2008), and with no obvious candidate genes to explore, a scan of the entire genome for associations with mathematical ability is desirable. Highly multiplexed microarrays permit such genome-wide coverage. However, the cost involved in individually genotyping the large sample sizes required to detect small QTL effects is prohibitive to most researchers. DNA pooling methods offer a possible solution. The DNA of multiple individuals may be combined and assayed on SNP microarrays to accurately detect differences between groups across the entire genome (Butcher *et al.* 2004, 2005a, b; Docherty *et al.* 2007; Meaburn *et al.* 2005; Pearson *et al.* 2007; Steer *et al.* 2007). Although individual genotyping remains the ultimate test of association, such pooling stages can be used to nominate sites for further investigation.

Here, we use pooled DNA from 10-year-olds of high vs. low mathematical ability (*N* = 600 each) in a two-stage GWAS of mathematical ability and disability. The top-performing 46 SNPs nominated in these two scanning stages were individually genotyped in a sample of 2356 individuals spanning the entire distribution of ability, to test not only the association with low vs. high mathematical performance, but also the QTL hypothesis that most SNPs associated with mathematical ability at the extremes are also associated with the entire range of mathematical ability.

## Materials and methods

### Participants

Participants were part of the Twins Early Development Study (TEDS), a longitudinal study involving a representative sample of over 11,000 sets of twins born in England and Wales between 1994 and 1996 (Oliver & Plomin 2007; Trouton *et al.* 2002). Comparisons to UK census data reveal that the TEDS sample continues to be representative of the UK population in terms of demographic characteristics (Harlaar *et al.* 2005). Throughout this study the sexes were analysed simultaneously to increase power, as quantitative genetic analyses have revealed neither qualitative nor quantitative sex differences in the genetic factors affecting mathematical ability (Kovas *et al.* 2007b; Oliver *et al.* 2004). We excluded children with specific medical syndromes such as Down's syndrome and other chromosomal anomalies, cystic fibrosis, cerebral palsy, hearing loss, autism spectrum disorder, organic brain damage, extreme outliers for birth weight, gestational age, maternal alcohol consumption during pregnancy, special care after birth, non-white ethnic origin (to mitigate population stratification), English spoken as second language at home (to facilitate a fair comparison of test performance scores) and those without DNA samples available. Following this, the sampling frame consisted of 5019 children selected on the basis of mathematics teacher ratings or web-assessed test data at age 10: 4077 with teacher ratings, 3918 with web-test data and 2976 twins with both.

### Measures

#### Composite measure

The selection of participants for this study was based on a composite measure of national curriculum-based teacher ratings and web-based mathematical tests for the 2976 children for whom both measures were available. Our multivariate genetic research indicates that these two types of measure are correlated phenotypically and genetically [0.53 and 0.62, respectively (Kovas *et al.* 2007b)] and combining them produces a more reliable measure for our GWAS. Each measure was standardized to a mean of zero and standard deviation of one. The mean of the two measures was then standardized to form the composite score. For an additional 1101 children, only teacher ratings were available and for 942 children only web-based measures were available. To increase the power of our sample to detect QTLs of small effect, these children were also included, with their one available score standardized to a mean of zero and standard deviation of one.

#### Web-based testing

The merits of web-based approaches have been well documented and findings appear consistent with traditional methods of data collection (Haworth *et al.* 2007). The battery used in this study included questions from three components of mathematics: ‘Understanding Number’, ‘Computation and Knowledge’ and ‘Non-Numerical Processes' (Kovas *et al.* 2007c) (see Supporting Information for a more detailed description). These components correspond to the UK National Curriculum (NC) and thus increase the relevance of the study to education. Battery items were based on the National Foundation for Educational Research 5–14 Mathematics Series, which is linked closely to curriculum requirements in the United Kingdom and the English Numeracy Strategy (nferNelson 2001). The results across the three categories were combined to generate a composite score of ability across the diverse domain of mathematics because multivariate genetic analyses reveal that the components are genetically highly correlated (Kovas *et al.* 2007c), suggesting that the genetic effects influencing ability across diverse areas of mathematics are general (Plomin & Kovas 2005). In the cohort from which the present sample was drawn, this general mathematics web-test score has yielded a heritability estimate of 0.49 (95% CI: 0.40–0.58) (Kovas *et al.* 2007a).

#### National curriculum-based teacher ratings

Mathematical ability was also measured by teachers' assessments on UK NC Key Stage 2 criteria for mathematical attainment (QCA 2001). The NC is a framework used by all government-maintained schools across the United Kingdom to ensure that teaching and learning is balanced and consistent. NC-based ratings therefore provide a reliable and uniform measure of mathematical ability across our sample. Teacher assessments have been revealed to be valid measures of academic achievement, particularly for mathematics, reading and language (Hoge & Coladarci 1989). The teachers assessed three aspects of mathematical ability: Using and applying mathematics; Numbers and algebra; and Shapes, space and measures (see Supporting Information and NC website for further details—http://curriculum.qcda.gov.uk/index.aspx). We created a mathematics composite mean score by summing standardized scores for the three ratings because our multivariate genetic analyses reveal that the ratings are highly correlated genetically (Kovas *et al.* 2007a), indicating that the genetic effects are general (Plomin & Kovas 2005). The heritability of this composite teacher-rating in the TEDS sample has been estimated as 0.64 (95% CI: 0.56–0.72) (Kovas *et al.* 2007a).

### Design and procedures

#### Stages 1 and 2: SNP microarrays and pooling (SNP-MaP) screen of low vs. high groups

In order to maximize the power of this DNA pooling study to detect associations of the small effect sizes expected here, a high- vs. low-ability design was employed (Jawaid *et al.* 2002). After collating the mathematics scores of the 5019 10-year-olds, a cut-off at the top and bottom 16th percentiles was used to select 300 individuals from the high extreme and 300 from the low extreme of performance for the first screening stage. A cut-off at the top and bottom 20th percentiles was used to select 300 high- and 300 low-performing individuals for the second screening stage. Only one member of a twin pair was selected within each screening stage—however sample 2 contained 73 monozygotic twins and 83 dizygotic twins of individuals from sample 1. Both screening stages followed the same design: within the high- and low-ability groups individuals were randomly allocated to one of 10 pools. Thus, 10 independent pools of mixed sex were created for each group, with each pool containing the DNA of 30 individuals. Genomic DNA for each individual was extracted from buccal swabs (Freeman *et al.* 2003), suspended in ethylenediaminetetraacetic acid (EDTA) TE buffer (0.01 m Tris-HCl, 0.001 m EDTA, pH 8.0) and quantified in triplicate using PicoGreen® dsDNA quantification reagent (Cambridge Bioscience, Cambridge, UK). Upon obtaining reliable quantification triplicate readings (±0.5 ng/µl), 100 ng of DNA for each individual was added to their respective pool. To avoid compromise because of pipetting errors 1 µl was deemed the minimum volume that could be reliably added to a pool. DNA samples found to be at a concentration greater than 100 ng/µl were diluted before being added to a pool. The range of final concentrations across the 20 pools for high and low mathematical ability was 14.77–16.13 ng/µl and 15.33–17.03 ng/µl, respectively.

#### SNP microarray allelotyping of pooled DNA

DNA pools were prepared for hybridization to GeneChip® Mapping 500K Arrays (Affymetrix, California, USA) in accordance with the standard protocol for individual DNA samples documented in the GeneChip® Mapping 500K Assay Manual. This platform has been previously validated for use with DNA pooling techniques (Docherty *et al.* 2007). In both pooling stages, each of the 20 pools, along with a reference DNA individual provided by the manufacturer (sample number 100103), was assayed on a separate microarray set. Each microarray was scanned using the GeneChip® Scanner 3000 with High-Resolution Scanning Upgrade and GeneChip® Operating software (GCOS) v1.4. Cell intensity (.cel) files were created using GeneChip® Genotyping Analysis Software (GTYPE v4.0). Relative allele signal (RAS) scores have been demonstrated as reliable and valid indices of allele frequency in pooled DNA (Butcher *et al.* 2004; Craig *et al.* 2005; Docherty *et al.* 2007; Kirov *et al.* 2006; Liu *et al.* 2005; Meaburn *et al.* 2005, 2006). Here, probe intensities were derived from the CEL files and combined to produce RAS scores using the SNP-MaP package (Davis *et al.* 2009) for the R statistical computing environment (R Development Core Team 2008). X-chromosome SNPs and SNPs with minor allele frequencies lower than 5% were removed from the analysis because of limited statistical power. SNPs were also removed because of poor performance in the WTCCC study using the same arrays (Wellcome Trust Case Control Consortium 2007). This left 358,948 autosomal SNPs for analysis. RAS scores from each probe quartet for these SNPs were analysed for association with high/low status using linear mixed-effects models in R (Bates & Sarkar 2006). High/low pool status was modelled as a fixed effect; array and assay strand were modelled as random effects. We did not attempt to derive true *P*-values for SNP associations from the pooling stages, which were intended as a screen of the genome to nominate SNPs for individual genotyping. Rather, estimated *P*-values were simply used to rank SNPs, and the 3000 top-ranked SNPs from the first stage were taken forward to the second stage, where the same analysis was used to select SNPs for stage 3.

#### Stage 3: Individual genotyping across the normal distribution

To validate the pooling results and to extend the analysis of the high and low extremes to a normally distributed population sample, the 46 top-ranked SNPs from the second stage were individually genotyped: 41 using the Sequenom MassARRAY iPlex Gold® system (Sequenom, San Diego, USA) and 5 using Applied Biosystems' TaqMan® assay (Applied Biosystems, California, USA). The medium-throughput Sequenom MassARRAY iPlex Gold® system processes ‘plexes’ of up to 40 SNP-assays simultaneously. Only compatible assays may be combined into a single plex. Because of this, and to economize on cost and man-hours, the 41 SNPs we investigated here using the Sequenom iPlex Gold® system were coupled with SNP-assays from other studies and spread across three plexes of 26, 33 and 36 SNPs. The sample for the individual genotyping stage comprised 2356 individuals (one member of each twin pair) drawn from a normal distribution of mathematics scores. 380 individuals within this sample overlapped with the 600 individuals in the second pooling stage. Of these 380 individuals, 66 had monozygotic twins and 78 had dizygotic twins within sample 1. The remainder of sample 3 also contained 142 monozygotic and 243 dizygotic twins of individuals from sample 1, and 82 monozygotic and 105 dizygotic twins of individuals in sample 2.

Individuals calling on fewer than 70% of the SNPs within each ‘plex’, and also within the TaqMan®-genotyped samples, were retyped, as were SNPs with a call rate lower than 95%. 22 individuals with persistently low call rates were removed entirely from the analysis leaving a final sample of 2334 individuals. However, on a ‘within-plex’ basis, 175, 172, 244 and 185 individuals were removed from the analysis of SNPs within the 26-plex, 33-plex, 36-plex and Taqman-genotyped SNPs, respectively. Three SNPs with persistently low call rates were also removed. The 43 remaining SNPs were assessed for Hardy—Weinberg equilibrium and analysed using linear models in R, fitting an additive model to test for association with mathematics, then testing for evidence of non-additivity by likelihood ratio test comparison of nested models (Balding 2006). The 10 significantly associated (*P* < 0.05) SNPs in sample 3 were then combined together to form a SNP-set score between 0 and 20 for each of the individuals in sample 3. This score equalled the number of mathematics-increasing alleles each individual possessed, and was analysed using linear models in R to gauge the effect of the SNP set on mathematics. Sample 3 was then dichotomized, and logistic regression used to test association of the 10 SNPs and the SNP-set score with low mathematical performance.

### Power

Power was estimated using the Genetic Power Calculator (Purcell *et al.* 2003). To account for the pooling procedure, estimates for the first two stages were based upon effective sample sizes of 68% the true size (Barratt *et al.* 2002). Using this criterion, under the additive model samples 1 and 2 had 80% power at the *P* < 0.05 level to detect a causal (i.e. *D*′ = 1.0 with a variant influencing mathematical ability) association with an allelic variant of 20% frequency and 1% and 1.25% effect size, respectively; with a marker in linkage disequilibrium (*D*′ = 0.8) with a causal variant of 20% frequency and 1.55% and 2% effect size, respectively. It is worth noting that *P*-values were used solely as a means of ranking SNP performance in the pooling stages. A threshold of *P* < 0.05 has been used simply to provide an estimate of the power of samples 1 and 2. With mathematical ability assessed as a quantitative trait under the additive model, at the *P* < 0.05 level the individual genotyping sample had 80% power to detect a causal variant of 20% frequency and 0.41% effect size; with a marker in linkage disequilibrium (*D*′ = 0.8) with a causal variant of 20% frequency and 0.65% effect size. The genotypic model provides the same power to detect non-additive effects, but has less power to detect additive effects. When dichotomized for case (defined as the lowest performing 15%) vs. control analyses, under the additive model at the *P* < 0.05 level the individual genotyping sample had 80% power to detect risk alleles for mathematical disability of 20% frequency and 0.75% effect size, and a marker in linkage disequilibrium (*D*′ = 0.8) with a causal variant of 20% frequency and 1.2% effect size.

## Results

**Figure 1:**
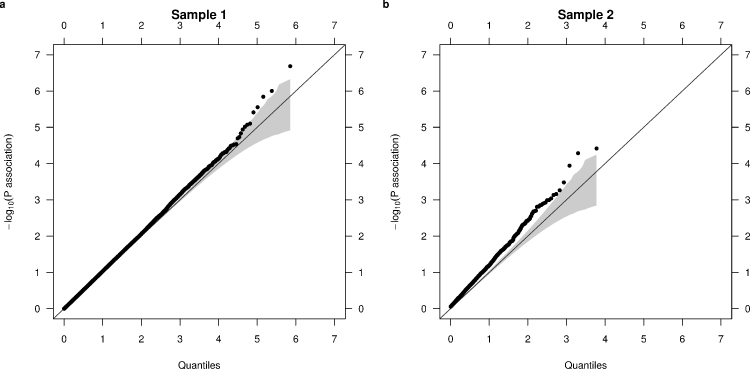
**Q-Q plot for samples 1 and 2.** Negative log base 10 *P*-values from a mixed-effects model likelihood ratio test are plotted against theoretical quantiles from the null distribution. The straight line at x = y represents the null distribution and the grey areas represent 95% bootstrapped confidence intervals on the null. Panel (a) includes all SNPs passing quality control assayed in sample 1 and reveals only slight deviation from chance association; panel (b) displays only the top 3000 SNPs from sample 1 tested in sample 2 (one-tailed). Deviation from expected is greater here, indicating an increase in the presence of true associations.

Figure 1 displays quantile—quantile plots of the ranked *P*-values obtained from comparing the pooled DNA of 300 individuals of high mathematical ability to that of 300 individuals of low mathematical ability in samples 1 and 2. Figure 1a includes *P*-values from sample 1 for all of the 358,948 SNPs assessed. Although a small number of SNPs rise above the identity line, few fall outside the bootstrapped 95% confidence intervals on the null hypothesis, suggesting only slight deviation from chance association. The top-performing 3000 SNPs from sample 1 were tested for association in the same direction in sample 2. Figure 1b displays one-tailed test *P*-values for these SNPs in sample 2. Although none of the SNPs reach genome-wide significance, a far greater deviation from expectation under the null hypothesis is demonstrated here, indicating a possible enrichment of true associations relative to all of the SNPs tested in the first scanning stage.

It should be noted that technical artefacts or population stratification might also result in such a deviation. Batch effects were avoided by the randomization and simultaneous processing of high/low mathematical ability pools within each scanning stage. Although we were unable to use conventional tests for population stratification in the pooling stages, strong stratification effects were not expected because participants were of the same ethnicity and drawn from a representative UK population-based sample. Furthermore, qq-plots revealed that the 12 autosomal ancestry-informative regions (the 13th X-chromosomal region was not analysed here) identified by the WTCCC as revealing strong geographical differentiation across the United Kingdom (Wellcome Trust Case Control Consortium 2007) did not associate with mathematical ability in either sample 1 or sample 2 (see Fig. S1), and none of the SNPs selected for individual genotyping from the two-stage scan fell within these ancestry-informative regions.

**Figure 2:**
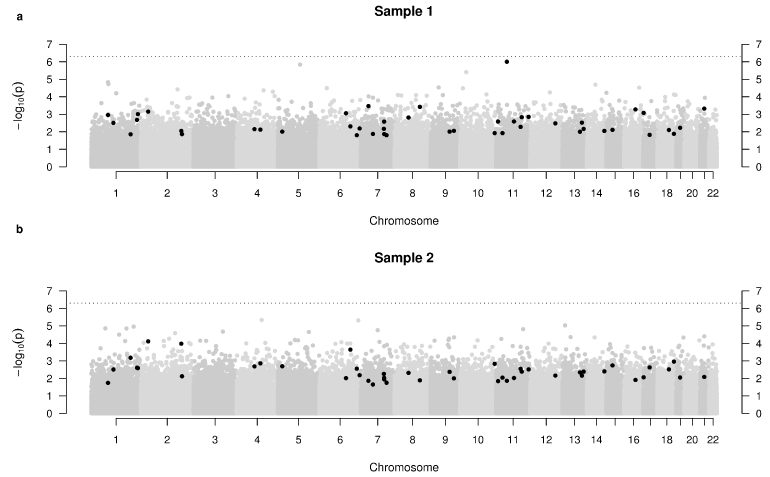
**Whole-genome plots of ***P*** -values obtained from samples 1 and 2.** Only those SNPs revealing between-group RAS differences in the same direction across both samples are plotted. Of the 3000 SNPs revealing the greatest differences in sample 1, the top-performing 46 in sample 2 were selected for further study. These SNPs are marked in black.

Figure 2 displays genomic plots of *P*-values from samples 1 and 2. Marked in black are the top-performing 46 SNPs from the 3000 SNPs tested in sample 2. For financial reasons, 46 was the maximum number of SNPs we could afford to carry forward to an individual genotyping stage. Figure 2 indicates that few of the 46 SNPs revealed the most extreme between-group differences in either sample, and so would have been overlooked in either one of the genome-wide scans alone. Table 1 provides more detailed results for these 46 SNPs in samples 1 and 2.

**Table 1:** Pooling results for the 46 top-performing SNPs in samples 1 and 2

			Sample 1					Sample 2				
SNP rsID	Chr	Position	Low RAS	High RAS	RAS diff	Chi	*P*-value	Low RAS	High RAS	RAS diff	Chi	One-tail *P*-value
rs952312	1	80256562	0.686	0.643	0.043	10.67	0.001	0.727	0.682	0.044	5.60	0.009
rs694598	1	103046767	0.672	0.628	0.045	8.75	0.003	0.697	0.660	0.037	8.73	0.002
rs6701879	1	204684389	0.620	0.664	−0.043	6.06	0.014	0.606	0.644	−0.038	11.58	3.3E –04
rs4649372	1	230646220	0.445	0.491	−0.046	9.48	0.002	0.404	0.451	−0.047	9.16	0.001
rs696244	1	233728167	0.564	0.513	0.051	10.85	0.001	0.549	0.499	0.050	9.07	0.001
rs1881396	2	27698105	0.377	0.320	0.057	11.47	0.001	0.377	0.315	0.062	15.63	3.8E –05
rs2059357	2	186034194	0.416	0.377	0.038	6.84	0.009	0.372	0.324	0.048	15.07	5.2E –05
rs12613365	2	191055555	0.434	0.463	−0.029	6.08	0.014	0.410	0.448	−0.038	7.12	0.004
rs1502885	4	79205538	0.582	0.633	−0.051	7.26	0.007	0.605	0.649	−0.044	9.49	0.001
rs4956093	4	108529657	0.570	0.509	0.061	7.14	0.008	0.576	0.507	0.068	10.22	0.001
rs17278234	5	13990476	0.551	0.592	−0.041	6.67	0.010	0.554	0.598	−0.044	9.53	0.001
rs7745469	6	110069819	0.535	0.581	−0.046	11.08	0.001	0.519	0.560	−0.041	6.69	0.005
rs11154532	6	130567068	0.525	0.593	−0.069	7.91	0.005	0.536	0.638	−0.102	13.58	1.1E –04
rs12199332	6	157185419	0.611	0.654	−0.044	5.85	0.016	0.620	0.678	−0.058	8.94	0.001
rs2278677	6	166495777	0.354	0.425	−0.071	7.42	0.006	0.284	0.341	−0.058	7.38	0.003
rs39118	7	29320557	0.380	0.437	−0.056	12.85	3.4E –04	0.356	0.394	−0.038	6.07	0.007
rs4236383	7	46856016	0.534	0.495	0.039	6.13	0.013	0.542	0.508	0.034	5.20	0.011
rs6947045	7	107287183	0.313	0.359	−0.047	7.33	0.007	0.306	0.343	−0.036	7.69	0.003
rs2300052	7	107875730	0.686	0.632	0.054	9.05	0.003	0.687	0.640	0.047	6.75	0.005
rs40941	7	107990161	0.569	0.521	0.048	6.10	0.013	0.568	0.526	0.041	6.41	0.006
rs7791660	7	122916256	0.657	0.623	0.034	5.84	0.016	0.686	0.654	0.033	5.62	0.009
rs11778957	8	54659415	0.512	0.462	0.051	10.05	0.002	0.523	0.473	0.050	7.93	0.002
rs10098370	8	105880919	0.693	0.638	0.055	12.66	3.7E –04	0.723	0.687	0.036	6.18	0.006
rs700965	9	97550959	0.322	0.373	−0.051	6.68	0.010	0.282	0.331	−0.049	8.19	0.002
rs4314720	9	112411728	0.324	0.358	−0.034	6.87	0.009	0.323	0.356	−0.033	6.65	0.005
rs7085203	10	130600913	0.690	0.664	0.026	6.33	0.012	0.713	0.674	0.039	10.12	0.001
rs7932127	11	7546172	0.589	0.525	0.063	9.07	0.003	0.563	0.524	0.039	6.00	0.007
rs16907131	11	20925249	0.717	0.677	0.040	6.33	0.012	0.772	0.735	0.037	6.82	0.005
rs10501162	11	36703331	0.310	0.380	−0.070	23.93	1.0E –06	0.293	0.329	−0.036	6.06	0.007
rs1369458	11	78438121	0.408	0.343	0.065	9.07	0.003	0.350	0.308	0.042	6.74	0.005
rs11225308	11	101904688	0.597	0.646	−0.050	7.81	0.005	0.565	0.628	−0.062	8.91	0.001
rs6588923	11	106125102	0.824	0.861	−0.037	10.12	0.001	0.795	0.829	−0.034	8.23	0.002
rs7115849	11	130149959	0.802	0.831	−0.028	10.20	0.001	0.782	0.814	−0.032	8.78	0.002
rs1215603	12	105041007	0.605	0.561	0.044	8.62	0.003	0.582	0.537	0.045	7.31	0.003
rs9670398	13	89692996	0.674	0.634	0.040	6.63	0.010	0.705	0.659	0.046	8.03	0.002
rs4771280	13	97136856	0.683	0.641	0.042	8.80	0.003	0.710	0.675	0.035	7.27	0.004
rs9300810	13	102806158	0.616	0.677	−0.061	7.29	0.007	0.650	0.697	−0.047	8.25	0.002
rs4144132	14	95761402	0.638	0.675	−0.037	6.84	0.009	0.625	0.675	−0.050	8.28	0.002
rs2593170	15	50010709	0.528	0.583	−0.055	7.09	0.008	0.549	0.606	−0.057	9.71	0.001
rs8043884	16	63140767	0.585	0.652	−0.067	12.01	0.001	0.595	0.627	−0.032	6.26	0.006
rs6502244	17	2300059	0.260	0.296	−0.036	11.17	0.001	0.243	0.280	−0.037	6.90	0.004
rs12601191	17	36361635	0.636	0.596	0.041	5.91	0.015	0.661	0.626	0.035	9.23	0.001
rs12962177	18	48464878	0.452	0.503	−0.051	7.05	0.008	0.444	0.485	−0.041	8.76	0.002
rs17085111	18	67590335	0.570	0.514	0.057	6.16	0.013	0.599	0.531	0.068	10.66	0.001
rs16964420	19	35802073	0.483	0.546	−0.063	7.57	0.006	0.473	0.542	−0.069	6.82	0.005
rs363449	21	29906146	0.635	0.572	0.063	12.21	4.8E –04	0.641	0.599	0.042	6.98	0.004

The 3000 top-performing SNPs in sample 1 were tested for association in sample 2 and the top-performing 46 were selected for individual genotyping in sample 3. The table is arranged by genomic location. SNP rsID, dbSNP rsID; Chr, chromosome; Position, physical position; Low/High RAS, relative allele signal score for the Affymetrix-assigned ‘allele A’ in low/high mathematics ability group. Broadly speaking RAS scores can be thought of as an estimate of allele frequency within a group; Chi, Chi-squared value obtained from a mixed-effects model likelihood ratio test with 1 degree of freedom; *P*-value, *P*-value obtained from a linear mixed-effects model likelihood ratio test. One-tail test because only associations in the expected direction are accepted as significant.

These 46 SNPs were tested for QTL association via individual genotyping in a third sample of 2356 individuals spanning the entire distribution of mathematical ability. Although SNPs with call rates below 95% were retyped, 3 of the 46 SNPs yielded persistently low call rates and were excluded from further analyses. 22 individuals with persistently low call rates were also completely excluded at this stage, leaving a sample of 2334 individuals. In our sample there was no significant effect of sex on either mathematical ability or its association with any of the remaining 43 SNPs; for this reason and to increase power the sexes were analysed together. All 43 SNPs were in Hardy—Weinberg equilibrium at the *P*> 0.01 level.

**Figure 3:**
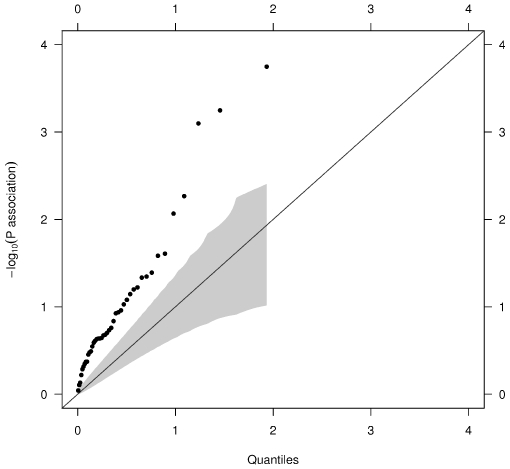
**Q-Q plot for mathematics association results of the 43 SNPs individually genotyped in sample 3.** Grey region indicates bootstrapped 95% confidence intervals. 10 SNPs reach nominal significance and 3 survive Bonferroni correction for multiple testing. The observed distribution deviates from the expected very early on, suggesting the presence of further associations which might be detected in a sample with more power.

Figure 3 displays a quantile—quantile plot of the *P*-values obtained for the 43 individually genotyped SNPs in sample 3, which demonstrates that more associations were observed than would be expected under the null hypothesis. The observed distribution deviates from the expected distribution very early on, suggesting that more associations of small effect size might be detected in a larger sample with more power. Table 2 contains detailed individual genotyping results. Under the additive model for association, 10 SNPs (rs11225308, rs363449, rs17278234, rs11154532, rs12199332, rs12613365, rs6588923, rs2300052, rs6947045 and rs1215603) remain significantly associated (*P* < 0.05) with individual differences in mathematical ability. Moreover, the first three associations in Table 2 remain significant after Bonferroni correction for all 43 SNPs tested. These intronic SNPs are located within *MMP7, GRIK1* and *DNAH5*, respectively (see Table 3).

**Table 3:** Additional analyses of individual genotyping data for 10 top-performing SNPs selected for mathematics ‘SNP set'

SNP rsID	Chr	Position	Allele	*N*	Additive model *F*	Additive model *P*	Genotypic model *F*	Genotypic model *P*	Non- additivity *F*	Non- additivity *P*	Case- control *P*	OR (CI)	Nearest gene
rs11225308	11	101904688	G	2179	12.78	0.0002	7.43	0.0003	2.07	0.1502	0.0001	1.43 (1.12–1.82)	MMP7
rs363449	21	29906146	C	2177	10.63	0.0006	5.43	0.0022	0.24	0.6231	0.0017	1.50 (1.03–2.25)	GRIK1
Rs17278234	5	13990476	C	2183	9.99	0.0008	5.05	0.0032	0.11	0.7395	0.0437	1.20 (0.94–1.53)	DNAH5
Rs11154532	6	130567068	C	2173	6.51	0.0054	3.45	0.0159	0.40	0.5264	0.0005	1.42 (1.11–1.81)	SAMD3
Rs12199332	6	157185419	A	2111	5.69	0.0086	4.13	0.0081	2.57	0.1094	0.2563	1.13 (0.88–1.46)	ARID1B
Rs12613365	2	191055555	G	2179	3.87	0.0246	1.95	0.0713	0.03	0.8640	0.2427	1.10 (0.67–1.89)	FLJ20160
Rs6588923	11	106125102	A	2111	3.78	0.0260	2.14	0.0590	0.50	0.4802	0.0663	1.26 (0.94–1.66)	GUCY1A2
Rs2300052	7	107875730	G	2109	3.04	0.0407	1.60	0.1011	0.16	0.6887	0.0863	1.42 (0.88–2.40)	NRCAM
Rs6947045	7	107287183	C	2179	2.87	0.0451	2.70	0.0338	2.52	0.1128	0.1803	0.96 (0.70–1.33)	DLD
Rs1215603	12	105041007	C	2179	2.83	0.0463	1.42	0.1212	0.01	0.9358	0.0543	1.25 (0.91–1.74)	NUAK1

The table is ordered by additive model *P*-value (one-tailed). The first three SNP associations survive Bonferroni correction for multiple testing. SNP rsID, dbSNP rsID; Chr, chromosome; Position, physical position; Allele, allele associated with lower mathematical ability in our sample; *N*, Sample *N*; Additive model *F*, *F*-statistic from the additive model on 1 and *N*− 2 degrees of freedom; Additive model P = one-tailed P value from additive model; % Variance = variance in mathematical ability explained by the additive model; Genotypic model F = F-statistic from the genotypic model on 1 and N − 3 degrees of freedom; Genotypic model *P*, one-tailed *P*-value from genotypic model; Non-additivity *F*, *F*-statistic from the additive vs. genotypic model comparison on 1 degree of freedom; Non-additivity *P*, *P*-value from likelihood ratio test comparison of additive and genotypic models; Case-control *P* = one -tailed *P*-value from logistic regression test (with 1 degree of freedom) of association with case status defined as the lowest-performing 15%; OR (CI), Odds ratio for case risk; Nearest gene, Nearest gene annotation from NetAffx (http://www.affymetrix.com/analysis/index.affx).

**Table 2:** Results for 43 SNPs individually genotyped in sample 3

SNP rsID	Chr	Position	Allele A	Allele B	MAF	N	Mean AA	Mean AB	Mean BB	F	Additive model *P*	% Variance
rs11225308	11	101904688	G	T	0.23	2179	−0.289	−0.037	0.047	12.78	0.0002	0.58
rs363449	21	29906146	C	G	0.38	2177	−0.072	0.012	0.141	10.63	0.0006	0.49
rs17278234	5	13990476	C	T	0.31	2183	−0.157	−0.031	0.062	9.99	0.0008	0.46
rs11154532	6	130567068	C	T	0.23	2173	−0.096	−0.062	0.047	6.51	0.0054	0.30
rs12199332	6	157185419	A	G	0.20	2111	−0.015	−0.093	0.044	5.69	0.0086	0.27
rs12613365	2	191055555	G	T	0.25	2179	−0.038	0.036	0.090	3.87	0.0246	0.18
rs6588923	11	106125102	A	G	0.12	2111	−0.253	−0.051	0.020	3.78	0.0260	0.18
rs2300052	7	107875730	A	G	0.30	2109	0.103	0.015	−0.032	3.04	0.0407	0.14
rs6947045	7	107287183	C	T	0.43	2179	−0.070	0.041	0.014	2.87	0.0451	0.13
rs1215603	12	105041007	C	T	0.44	2179	−0.046	0.008	0.055	2.83	0.0463	0.13
rs40941	7	107990161	C	T	0.35	2101	−0.053	0.057	−0.004	2.42	0.0599	0.12
rs1881396	2	27698105	G	T	0.21	2181	−0.070	−0.037	0.026	2.33	0.0634	0.11
rs4649372	1	230646220	A	T	0.25	2180	0.181	−0.015	−0.014	2.15	0.0716	0.10
rs2593170	15	50010709	C	T	0.41	2168	−0.044	−0.011	0.039	1.92	0.0832	0.09
rs9300810	13	102806158	C	G	0.17	2112	0.017	−0.027	−0.111	1.74	0.0937	0.08
rs4314720	9	112411728	A	G	0.21	2177	−0.018	0.021	0.088	1.51	0.1097	0.07
rs39118	7	29320557	A	C	0.11	2182	−0.015	0.055	0.017	1.43	0.1157	0.07
rs694598	1	103046767	A	T	0.21	2104	0.003	0.041	−0.028	1.39	0.1189	0.07
rs11778957	8	54659415	A	G	0.39	1937	0.047	−0.047	0.012	1.11	0.1458	0.06
rs7085203	10	130600913	C	T	0.14	2180	−0.018	0.065	−0.087	0.88	0.1743	0.04
rs9670398	13	89692996	A	T	0.24	2138	0.107	0.002	−0.006	0.80	0.1851	0.04
rs4956093	4	108529657	A	C	0.39	2148	−0.020	0.011	0.030	0.72	0.1987	0.03
rs2278677	6	166495777	C	T	0.10	2111	−0.006	0.014	0.228	0.66	0.2088	0.03
rs16964420	19	35802073	A	G	0.31	2084	−0.070	0.008	0.011	0.64	0.2127	0.03
rs16907131	11	20925249	A	G	0.06	2175	0.049	0.045	−0.005	0.56	0.2274	0.03
rs7932127	11	7546172	C	T	0.27	2176	0.161	−0.032	0.004	0.54	0.2307	0.02
rs4236383	7	46856016	C	T	0.34	2100	−0.004	0.026	−0.027	0.54	0.2313	0.03
rs4144132	14	95761402	C	T	0.17	2149	−0.085	0.008	0.019	0.53	0.2341	0.02
rs10098370	8	105880919	A	G	0.18	2112	0.270	−0.030	0.000	0.48	0.2449	0.02
rs6502244	17	2300059	C	T	0.13	2176	0.061	0.017	−0.009	0.43	0.2568	0.02
rs6701879	1	204684389	C	T	0.19	2112	−0.085	−0.003	0.006	0.33	0.2836	0.02
rs1502885	4	79205538	A	T	0.17	2138	−0.311	0.057	−0.002	0.21	0.3235	0.01
rs4771280	13	97136856	C	G	0.28	2178	−0.001	−0.006	0.055	0.19	0.3324	0.01
rs7791660	7	122916256	C	T	0.19	2064	−0.002	−0.002	0.085	0.14	0.3526	0.01
rs8043884	16	63140767	A	C	0.28	2180	0.019	−0.013	0.007	0.04	0.4249	0.00
rs17085111	18	67590335	C	T	0.37	2180	0.036	−0.027	0.018	0.03	0.4264	0.00
rs700965	9	97550959	C	T	0.33	2109	−0.017	0.030	−0.057	0.02	0.4495	0.00
rs7115849	11	130149959	A	G	0.08	2181	−0.116	0.004	0.000	0.00	0.4823	0.00
rs1369458	11	78438121	A	G	0.13	2087	0.004	−0.006	0.102	0.00	0.5187	0.00
rs7745469	6	110069819	C	T	0.38	2097	0.008	0.005	−0.006	0.07	0.6023	0.00
rs952312	1	80256562	C	T	0.08	2112	0.175	−0.062	0.008	0.41	0.7400	0.02
rs12962177	18	48464878	C	T	0.48	2109	0.029	−0.010	−0.019	0.63	0.7861	0.03
rs2059357	2	186034194	C	G	0.25	2082	0.029	−0.017	−0.070	1.76	0.9076	0.08

The table is ordered by additive model *P*-value (one-tailed), the light grey area highlights the nominally significant SNPs and the darker grey area highlights those SNP associations withstanding bonferroni correction for multiple testing. SNP rsID, dbSNP rsID; Chr, chromosome; Position, physical position; MAF, minor allele frequency; *N*, sample *N*; Mean AA, mean quantitative trait mathematics scores for individuals with AA genotype; *F*, *F*-statistic from the additive model on 1 and *N*− 2 degrees of freedom; Additive model *P*, one-tailed *P*-value from additive model; % Variance, variance in mathematics ability explained by the additive model.

**Figure 4:**
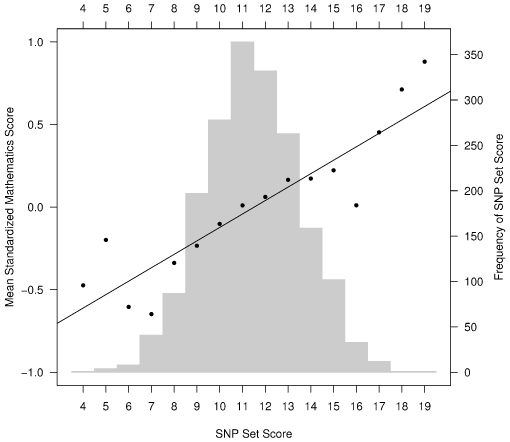
**Correlation between SNP-set score and mathematical ability.** SNP-set scores were created for individuals in sample 3 by summing performance-increasing allele scores across the 10 associated SNPs identified—rs11225308, rs363449, rs17278234, rs11154532, rs12199332, rs12613365, rs6588923, rs2300052, rs6947045 and rs1215603. Here, average math scores are plotted against SNP-set scores. Grey bar chart demonstrates the number of individuals with each SNP-set score. The graph runs only from 4 to 19 as there were no individuals with SNP-set scores of 0 to 3 or 20. When association between SNP-set score and mathematical performance was tested across all individuals in sample 3 using linear models, the SNP-set was found to account for 2.9% (*F* = 56.85; d *f* = 1 and 1881; *P* = 7.277e–14) of the variance in mathematics score—i.e. a correlation of 0.17.

The effect sizes of these 10 SNPs are small—with the largest at rs11225308 accounting for 0.58% of the variance in ability in the sample and the smallest at rs1215603 accounting for only 0.13%. However, when all 10 SNPs are combined in an additive model to form a ‘SNP set', together they account for 3.4% (*F* = 6.52; df = 10 and 1872; *P* = 5.766e–10; *N* = 1883) of the phenotypic variance. This method takes into account the relative effect sizes of each SNP in our sample and weights them in the model accordingly. As our sample may not have the power to accurately distinguish the exact magnitude of such small effect sizes, we also created a ‘SNP-set score’ for each individual in sample 3 by summing the math-increasing alleles they possess. This SNP-set score, in which each SNP is weighted equally, accounts for 2.9% (*F* = 56.85; df = 1 and 1881; *P* = 7.277e–14; *N* = 1883; see Fig. 4) of the variance in math ability in our sample. Figure 4 suggests that the relationship is linear across the distribution of mathematical ability, consistent with the QTL hypothesis. We also investigated the association of this 10-SNP set with the two component measures of the mathematics composite score. Although sample sizes were reduced, the SNP set accounted for 2.2% of the variance in teacher ratings (*F* = 34.47; df = 1 and 1531; *P* = 5.302e–09; *N* = 1533) and 2.1% of the variance in web-test performance (*F* = 31.96; df = 1 and 1484; *P* = 1.883e–08; *N* = 1486). When the same method was used to calculate a 43-SNP-set score from all SNPs tested in sample 3, we found it accounted for 3.2% (*F* = 46.18; df = 1 and 1380; *P* = 1.600e–11; *N* = 1382) of the variance in mathematics composite score in our sample. Figure 3 indicates the presence of true associations of effect sizes too small to be detected with the power of our sample.

Table 3 displays the results of further investigation of the 10 SNPs reaching nominal significance. After replicating these associations under the additive model, a likelihood ratio test of nested models was conducted to assess any possible non-additivity in their action. If cognitive capacities have been subject to directional selection, additive variance would be eroded leaving dominance variance unaffected, which would result in non-additive associations (Crnokrak & Roff 1995). None of the10 SNP associations identified here reveal significant non-additive action, although with such small effect sizes power is limited to distinguish between additive and non-additive models. Moreover, this result does not disprove directional selection because the SNPs were nominated in the first two stages under an additive model based on allele frequencies; also, genome-wide association studies of this sort are likely to detect indirect association with SNPs in linkage disequilibrium with functional variants (Donnelly 2008).

**Figure 5:**
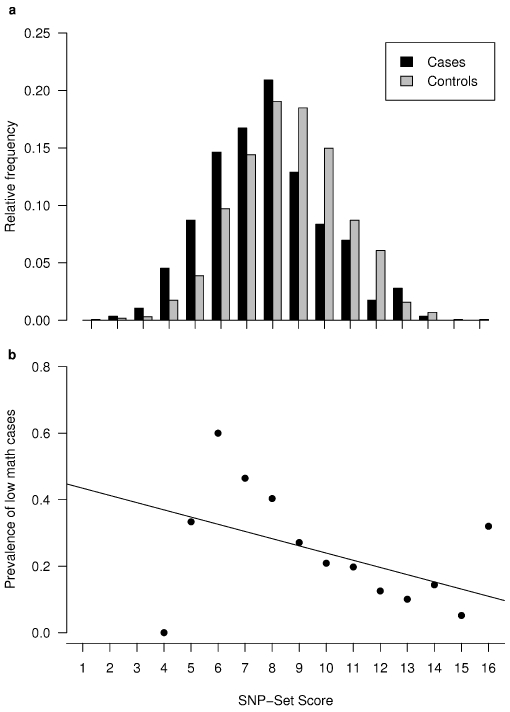
**Relationship of SNP-set score to prevalence ratio of low mathematical performance (defined as the lowest performing 15%).** SNP-set scores were gauged by summing the performance-increasing allele scores across the 10 associated SNPs identified—rs11225308, rs363449, rs17278234, rs11154532, rs12199332, rs12613365, rs6588923, rs2300052, rs6947045 and rs1215603. The graph runs only from 4 to 19 as there were no individuals with SNP-set scores of 0 to 3 or 20. Plot displays (a) relative frequencies of SNP-set scores within case (low mathematical performance) and control groups and (b) the relationship of SNP-set score to prevalence of cases of low mathematical performance. Analysis across all individuals in sample 3 revealed the SNP-set to be significantly associated with cases of low mathematical performance (*P* = 4.18e –09; df = 1881; N = 1883).

In addition to the analysis of mathematical ability as a continuous trait, sample 3 was dichotomized to assess the effect of these 10 SNPs on the lowest performing 15% of the sample. Although power is reduced in this approach, 4 of the 10 SNPs revealed significant associations with low performance (Table 3). The 10-SNP-set score was highly significantly associated with low performance (*P* = 4.18e − 09; df = 1881; N = 1883), and individuals within our sample with 10 or more of the 20 risk alleles were nearly twice as likely as those with 9 or fewer risk alleles to fall within the low performance group (OR = 1.96; 95% confidence intervals = 1.50–2.55; df = 1; *P* = 3.696e − 07; N = 1883). Figure 5 displays the results, which confirm the effect on low performance suggested in Fig. 4. A Kolmogorov—Smirnov test reveals that the distribution of risk alleles carried is significantly different in the case and control populations (D = 0.1753; *P*-value = 6.477e–07). The proportion of cases falls as the SNP-set score (i.e. the number of performance-increasing alleles) rises. The sensitivity and specificity of the SNP set to identify a child as low performing is 0.46 and 0.88, respectively, which translates to a positive predictive value (PPV) of 0.46 and a negative predictive value (NPV) of 0.70 within our sample.

Finally, we analysed sample 3 with the overlapping 380 (16%) individuals from sample 2 removed. Of the 10 SNP associations reported, those of rs11225308, rs363449, rs12199332 and rs17278234 remain nominally significant after this exclusion (*P* < 0.05, data not shown). A SNP-set score combining these four SNPs accounts for 1.2% of the variance in mathematical ability in this smaller sample (*F* = 19.21; df = 1 and 1594; *P* = 1.246e–05; *N* = 1596). Individuals with four or more of the eight risk alleles are nearly 1.5 times as likely to fall within the lowest performing 15% of the sample (OR = 1.39; 95% CI = 1.02–1.90; df = 1; *P*-value = 0.031; *N* = 1596; PPV = 0.18; NPV = 0.86).

## Discussion

This first GWAS of mathematical ability and disability has nominated 46 SNP associations across two high- vs. low-ability samples, 10 of which have been validated in a third sample spanning the entire distribution of ability as a test of the QTL hypothesis. As we report no large effects, our results are compatible with those of studies of other cognitive abilities (Butcher *et al.* 2005a, 2008; Meaburn *et al.* 2007) and complex traits (McCarthy *et al.* 2008), and suggest that genetic influence on mathematical ability is caused by multiple QTLs of small effect. Even so, when combined into a set, the 10 SNPs account for 2.9% of the phenotypic variance in our sample. The nomination of this set of SNPs in two high- vs. low-ability samples, and the significant influence the set demonstrates over individual differences across the normal distribution of ability, supports the QTL hypothesis that the same genes affect the entire spectrum of phenotypic expression. The QTL hypothesis is bolstered further by the findings that the 10-SNP set demonstrates a linear association with mathematics scores across the distribution (Fig. 4), and that children in our sample with 10 or more of the 20 risk alleles are nearly twice as likely to be in the low-performing group. The 10-SNP set has some predictive value for low mathematical performance in our sample (PPV = 0.46, NPV = 0.70). With no large effects expected, if future research in larger, more highly powered independent samples can replicate and add to our findings, there may come a time when such a SNP set will be useful in predicting genetic risk for mathematical difficulties or genetic precocity.

The main limitation of this study is power. Although the sample size was large, its power is limited to detect SNP associations of the small effect size that emerged from the GWAS. The pooling approach used to nominate SNP associations reduced power further (Barratt *et al.* 2002). This is reflected in the fact that genome-wide significance levels were not reached in the two-stage scan. Although the addition of the second scanning stage improved the SNP-selection process, and ensured co-twins were in different samples, performing a joint analysis of samples 1 and 2 would have increased power (Skol *et al.* 2006). Nevertheless, the economical pooling method retained 80% power to detect QTLs of 1% and 1.25% effect sizes in samples 1 and 2, respectively, and nearly a quarter of SNPs selected from these samples replicated with QTL associations in the individually genotyped sample 3. Still, as Fig. 3 suggests a presence of additional associations of small effect sizes that sample 3 is underpowered to detect, further investigation in more highly powered samples is desirable.

The creation of a composite mathematics score from teacher ratings and web-test results represents a second limitation. However, as these two component measures were highly phenotypically and genetically correlated, and as we have reported equally strong 10-SNP-set associations for both measures, we believe this was a valid way to increase our sample size. Another issue concerns the large number of false positive results expected when conducting multiple tests on a genome-wide level. Our two-stage design was intended to go some way towards dealing with this problem. However, there are still many SNPs exhibiting RAS differences in one or both of our first two samples that may reflect true associations, yet have not been further investigated here because of financial restrictions. We have not corrected any of the *P*-values obtained from the pooled samples for the number of tests performed. This is because these first two stages were intended simply as a means of screening SNPs for inclusion into the individual genotyping stage of our design. The *P*-values were used only to rank SNPs in the first two pooling stages. As multiple-testing correction would not alter the rank order of the SNPs, it would not have affected the outcome of these screening stages.

Another limitation is the overlap of *n* = 380 between samples 2 and 3. As the exclusion of these individuals greatly depletes the extremes—and therefore the statistical power—of sample 3, and as these overlapping individuals' genotypes and quantitative trait scores for mathematics is new information which was lost in the pooling stage, we decided not to exclude them from the main analysis. However we did re-run all analyses on the smaller sample, with some promising results. In addition to this direct overlap, 156 sample 1 individuals have co-twins in sample 2, and 716 sample 3 individuals have co-twins in samples 1 or 2. As this may positively bias our findings by over-inflating *P*-values it is one of the most important limitations of the study, however when striving for the largest possible *N* from a twin sample, such an overlap was unavoidable.

Although the three samples were not completely independent, information gathered from samples 1 and 2 concerned allelic and mathematical-performance group averages, and was used solely to identify SNPs for testing in a third sample comprising only one twin of a pair, using individual genotypic and phenotypic information. Nevertheless, we have selected and then tested SNPs in samples which overlap entirely in the phenotypic measures used, and also to a large extent genetically. Although this matching of measures and sample demographics overcomes many of the problems faced in the replication of molecular genetic findings, it also limits the ability to generalize our findings to a wider population. Further investigation of these SNPs in independent samples with greater statistical power is vital before we can draw any definite conclusions regarding their contributions to mathematical ability and disability. Indeed, our findings will almost certainly be subject to a ‘winner’s curse’ effect [discussed in Newton-Cheh & Hirschhorn (2005) and Kraft (2008)], in which the already small effect sizes reported have actually been overestimated in our discovery sample. The future of molecular genetic investigation into mathematics will ideally involve far larger, more highly powered samples to detect the expected small effects.

Although none of the SNPs identified fall within coding regions, or any known binding/splicing sites of interest, they can contribute to a SNP set of potential markers for mathematical ability and disability. They may also highlight possible candidate genes for mathematical ability and disability (Table 3). One example is that of *NRCAM*, a gene encoding the Bravo/NrCAM neuronal cell adhesion molecule (Grumet M, 1997), a protein involved in neuron-neuron connections in the developing and mature nervous system, and implicated in synaptic plasticity and memory processes (Hoffman 1998).

In addition to this involvement in brain function, *NRCAM* has been reported to be associated with autism (Bonora *et al.* 2005; Marui *et al.* 2008). Although the intronic SNP implicated here (rs2300052) has not been studied in relation to autism, it is in high LD (*r*^2^ > 0.70) with all previously associated *NRCAM*-tagging SNPs (Bonora *et al.* 2005; Marui *et al.* 2008) based on HapMap data (The International HapMap Consortium 2007). Of the SNPs previously associated with autism, rs2300052 is in highest LD (*r*^2^ = 0.83) with rs2300045. Common haplotypes estimated from HapMap data indicate an association between the rs2300052 allele conferring lower mathematical ability and the rs2300045 allele conferring autism risk. This is in keeping with the observation that although some autistic savants exhibit high mathematical ability, autism is generally associated with lower IQ, and even within high-functioning individuals with Asperger's syndrome, mathematical ability is significantly lower (Chiang & Lin 2007). Although some studies reject *NRCAM* as an autism candidate (Hutcheson *et al.* 2004), our data suggest a possible link between low mathematical performance and autism risk through *NRCAM* function, although effects are likely to be small.

Of particular interest are *MMP7, GRIK1* and *DNAH5*, the genes associated with the top three ranking SNPs in our study, whose associations remained significant after Bonferroni correction for multiple testing. *MMP7* encodes a member of the matrix metalloproteinase (MMP) family. MMPs are involved in the breakdown of extracellular matrix during normal physiological processes such as embryonic development, growth and tissue repair (Chakraborti *et al.* 2003). *GRIK1* encodes an ionotropic glutamate receptor kainate 1. Kainate receptors mediate neurotransmission and synaptic plasticity (Bortolotto *et al.* 1999; Huettner 2003), and dysfunction has been implicated in a number of psychiatric phenotypes (Gratacòs *et al.* 2008; Woo *et al.* 2007). *DNAH5* encodes the dynein axonemal heavy chain 5 protein. Dynein is the force-generating component of cilia, the correct functioning of which is essential in all areas of embryonic growth (Hornef *et al.* 2006), and *DNAH5* in particular has been demonstrated as vital for normal brain development (Ibanez-Tallon *et al.* 2004).

The intricate involvement of these genes in development—especially the direct links of *GRIK1* and *DNAH5* to brain development—is indicative of the variety of genes one might expect to exert small effects over cognitive abilities such as mathematics. It is likely then that the influence of such genes would also be evident in other cognitive domains. Indeed, quantitative genetic research indicates a substantial genetic overlap between reading, mathematical and general cognitive ability (g) (Kovas *et al.* 2005; Markowitz *et al.* 2005). Although the 10 QTL associations identified here neither fall within previously reported dyslexia linkage regions (McGrath *et al.* 2006; Paracchini *et al.* 2007), nor overlap with findings of association studies of reading (Meaburn *et al.* 2007; Seshadri *et al.* 2007) and g (Butcher *et al.* 2005a, 2008), there may still be an overlap in their influence. Along with the essential replication of our results in large independent samples, one interesting future direction may be to explore the generalist genes hypothesis at the molecular genetic level, by investigating the effects of these SNPs on other cognitive abilities.

## Addendum

Corrections added after publication 16 December 2009.

The authors would like to correct the sample size of the study population from the original reported value of “2449” to “2356”. Due to this change, the following values on the respective pages have been updated accordingly.

Page 234, in the Abstract:

The sample size “*2449*” has been corrected to “*2356*”.

Page 235, in the Introduction:

The sample size “*2449*” has been corrected to “*2356*”.

Page 236, under Materials and Methods, in the section “Stage 3: Individuals Genotyping across the normal distribution”:

The sample size “*2449*” has been corrected to “*2356*”.

The sentence ”*126 individuals with persistently low call rates were also completely excluded at this stage, leaving a sample of 2323 individuals*.” has been revised to “*22 individuals with persistently low call rates were also completely excluded at this stage, leaving a sample of 2334 individuals*”.

The sentence ”*However, on a ‘within-plex’ basis, 268, 265, 337 and 278 individuals were removed from the analysis of SNPs within the 26-plex, 33-plex, 36-plex and Taqman-genotyped SNPs, respectively*.” has been revised to “*However, on a ‘within-plex’ basis, 175, 172, 244 and 185 individuals were removed from the analysis of SNPs within the 26-plex, 33-plex, 36-plex and Taqman-genotyped SNPs, respectively*”.

Page 236, under Materials and Methods, in the section ”Power”.

”*0.4% effect size*” has been changed to “*0.41% effect size*“

”*2% effect size*” has been changed to ”*1.2% effect size*”.

Page 237, under Results:

The sample size ”*2449*” has been corrected to ”*2356*.

The sentence ”*126 individuals with persistently low call rates were also completely excluded at this stage, leaving a sample of 2323 individuals*.” has been revised to ”*22 individuals with persistently low call rates were also completely excluded at this stage, leaving a sample of 2334 individuals*”.

Page 240, under Results:

The sentence ”*Finally, we analysed sample 3 with the overlapping 380 (15%) individuals from sample 2 removed*”. has been revised to ”*Finally, we analysed sample 3 with the overlapping 380 (16%) individuals from sample 2 removed*”.

## Supporting Information

Figure S1: QQ-plots for association of ancestry-informative regions with high/low mathematical ability in samples 1 and 2. Negative log base 10 *P*-values from a mixed-effects model likelihood ratio test are plotted against theoretical quantiles from the null distribution. The straight line at *x* = *y* represents the null distribution and the grey areas represent 95% bootstrapped confidence intervals on the null. *P*-values from (a) sample 1 and (b) sample 2 are plotted for the 784 SNPs represented on the Affymetrix 500K two-chip array set which are located within the 12 autosomal ancestry-informative regions identified by the Wellcome Trust Case Control Consortium as demonstrating strong geographical differentiation across the United Kingdom. The lack of association in these regions indicates no effect of population stratification in samples 1 or 2.

As a service to our authors and readers, this journal provides supporting information supplied by the authors. Such materials are peer-reviewed and may be re-organized for online delivery, but are not copy-edited or typeset. Technical support issues arising from supporting information (other than missing files) should be addressed to the authors.
