# Supplementary material for: A genome-wide association study identifies multiple loci associated with mathematics ability and disability
Source: Genes Brain Behav. 2010 Mar;9(2):234–47. doi: 10.1111/j.1601-183X.2009.00553.x (PMC2855870; doi:10.1111/j.1601-183X.2009.00553.x)

Supplementary Figure 1: QQ-plots for association of ancestry informative regions with high/low mathematical ability in samples 1 and 2. Negative log base 10 P values from a mixed-effects model likelihood ratio test are plotted against theoretical quantiles from the null distribution. The straight line at x = y represents the null distribution and the grey areas represent 95% bootstrapped confidence intervals on the null. P-values from (a) sample 1 and (b) sample 2 are plotted for the 784 SNPs represented on the Affymetrix 500K two-chip array set which are located within the 12 autosomal ancestry informative regions identified by the Wellcome Trust Case Control Consortium as showing strong geographical differentiation across the UK. The lack of association in these regions indicates no effect of population stratification in sample 1 or 2.


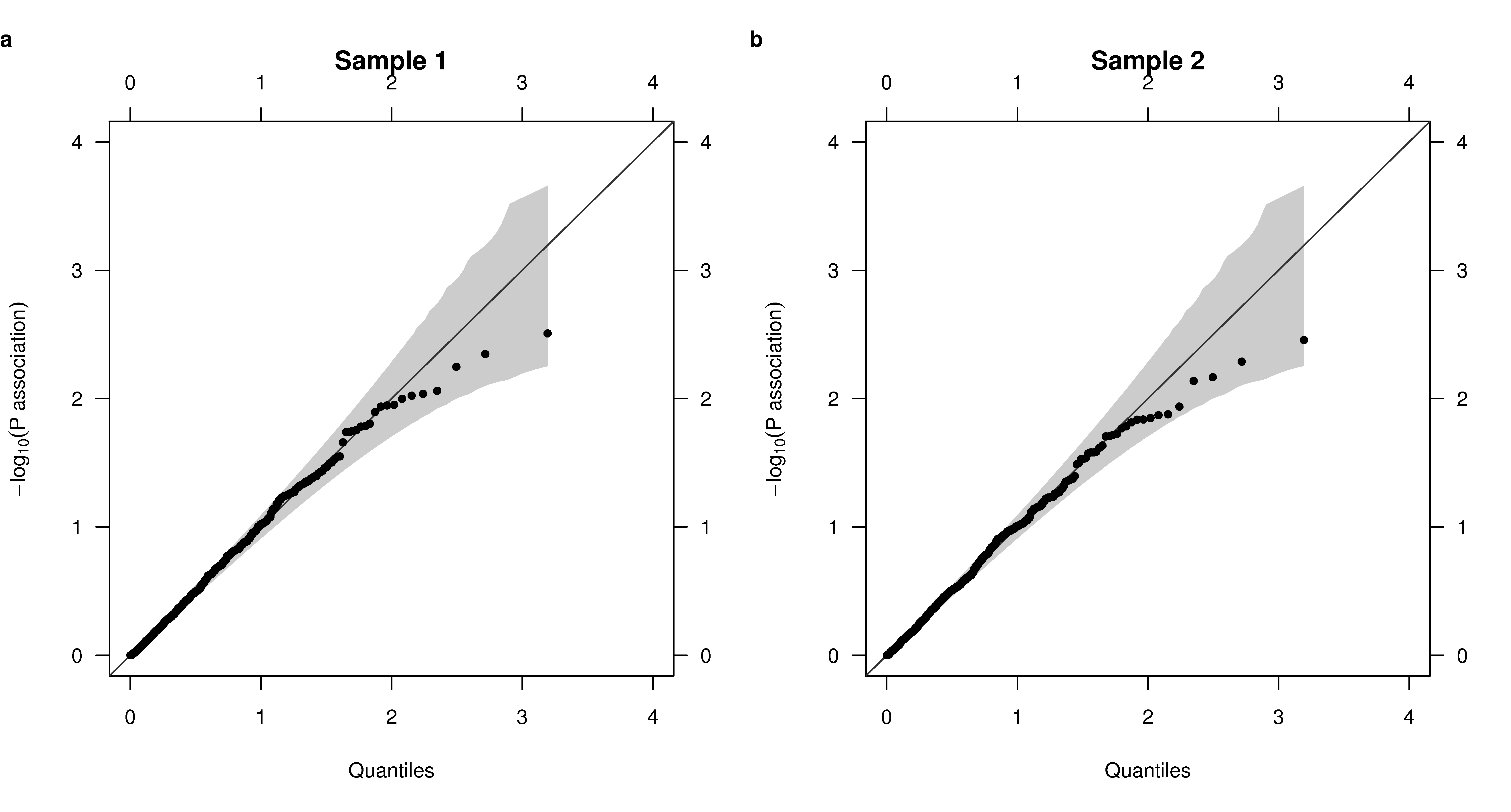

Supplement: Supplementary file 1 [file gbb0009-0234-SD1.doc]
